# X chromosome inactivation in human parthenogenetic embryonic stem cells following prolonged passaging

**DOI:** 10.3892/ijmm.2014.2044

**Published:** 2014-12-18

**Authors:** QUAN QI, CHENHUI DING, PINGPING HONG, GANG YANG, YANXIN XIE, JING WANG, SUNXING HUANG, KE HE, CANQUAN ZHOU

**Affiliations:** Reproductive Medical Center, The First Affiliated Hospital of Sun Yat-Sen University, Guangzhou, Guangdong 510080, P.R. China

**Keywords:** human parthenogenetic embryonic stem cells, karyotype, X chromosome inactivation, real-time PCR, X-inactive specific transcript RNA

## Abstract

The present study aimed to investigate the X chrochromosome inactivation (XCI) status in long-term cultured human parthenogenetic embryonic stem cells. One human embryonic stem (hES) cell line and 2 human parthenogenetic embryonic stem (hPES) cell lines were subjected to long-term culture *in vitro* (>50 passages). Karyotyping, array-based comparative genomic hybridization (aCGH), X-inactive specific transcript (XIST) RNA, immunofluorescence staining and real-time PCR were used to assess the chromosome karyotypes of these cells and the XCI status. X chromosome microdeletion was observed in the hPES-2 cells following culture for 50 passages. As early as 20 passages, XIST RNA expression was detected in the hPES-2 cells and was followed by low X-linked gene expression. The XIST RNA expression level was higher in the differentiated hPES-2 cells. The hPES-2′ cells that were subclones of hPES-2 retained the XCI status, and had low XIST and X-linked gene expression. XIST RNA expression remained at a low level in the differentiated hPES-2′ cells. The human biparental embryonic stem (hBES)-1 and hPES-1 cells did not exhibit XCI, and the differentiated hPES-1 cells had high expression levels of XIST RNA. In conclusion, the chromosome karyotypes of some hPES cell lines revealed instabilities. Similar to the hES cells, the hPES cells exhibited 3 XCI statuses. The unstable XCI status of the hPES-2 line may have been related to chromosome instability. These unstable chromosomes renedered these cells susceptible to environmental conditions and freezing processes, which may be the result of environmental adaptations.

## Introduction

Human embryonic stem (hES) cells have been widely used in regenerative medicine due to their capacity to differentiate into various cell types both *in vitro* and *in vivo* (1,2). However, transplant rejection and the unstable epigenetic state of hES cells from human embryos limit their use in research and therapy. Human parthenogenetic embryonic stem (hPES) cells, the genetic materials of which are derived entirely from a single oocyte, are considered to be a possible means to resolve the issue of immune rejection (3), and several hPES cell lines have been generated (4–8). These stem cell lines have exhibited infinite proliferation, self-renewal and differentiation properties, similar to embryonic stem cell lines *in vitro*.

The completely undifferentiated status and the original genetic characteristics of hES cells are important for clinical and research trials (9). It has been previously reported that the evolution and selection of hES cell clones cultured *in vitro* causes genetic and epigenetic changes, which alters the behavior and fate of these hES cells (10–13). The genetic and epigenetic stabilities of hES cells are crucial for their use in regenerative medicine. Epigenetic changes include DNA methylation, histone modifications, genomic imprinting and X chromosome inactivation (XCI).

XCI involves one of the X chromosomes in cells of a female mammal and is crucial for embryo formation and cell biology (14). To date, hES cells have been shown to have 3 different XCI statuses. With status I in the hES cells, both X chromosomes are activated in the undifferentiated stage, and XCI occurs randomly following differentiation, which is close to what occurs in mouse embryonic stem cells (15–17). With status II, XCI has already occurred in undifferentiated hES cells, and approximately 20–70% of hES cells can be found with X-inactive specific transcript (XIST) clouds accumulated on a specific chromosome (11,18). Finally, with status III, XCI has occurred without XIST RNA expression (11). Certain studies have demonstrated that the hES cell XCI states are related to the culture conditions used and spontaneous differentiation potential (19). However, the XCI statuses of hPES cell lines have not been thoroughly investigated to date.

Thus, in the present study, we assessed the statuses of hPES cell lines following prolonged passaging in culture *in vitro*. We focused on the XCI status of hPES cell lines (hPES-1 and hPES-2) under long-term culture conditions (>50 passages) *in vitro*. We found that hPES cells also had 3 XCI statuses, although different XCI statuses could be found within the same cell line. These differences in XCI status in hPES cells may be related to their X chromosome instability. Furthermore, low expression levels of X-linked genes were detected in the hPES cells that were related to their XIST RNA expression levels. The XCI status is related to the genetic characteristics or strains of embryonic stem cells that are cultured *in vitro* and the freezing conditions used. Our findings suggest that it is essential to assess the XCI status of hES cells and to consider this as one of the indicators used for evaluating the quality of hES cells.

## Materials and methods

### Ethics statement

Our protocols were approved by the Ethics Committee of the First Affiliated Hospital of Sun Yat-Sen University. Donors voluntarily donated experimental materials with no financial compensation and written informed consent was obtained.

### Derivation and culture of hES, human foreskin fibroblasts (HFFs) and human endometrial stromal cells (hESCs)

Three hES lines were analyzed in this study, including human biparental embryonic stem cell line-1 [hBES-1, passage (P)12], hPES cell line-1 (hPES-1, P10) and hPES cell line-2 (hPES-2, P10). The hBES-1 cells were from a cHES1 cell line that was derived and propagated in our embryonic stem cell laboratory, as previously described (20). The hPES-1 and hPES-2 cells were from hPES1 and hPES2 cell lines that were also derived and propagated in our laboratory, as previously described (6). Culture, cryopreservation and warming methods for undifferentiated hESCs and embryoid body (EB) formation were as previously described, and the origins and detailed characterizations of the pluripotency of these cell lines were verified (6,20,21). The derivation and culture of hESCs and HFFs were as previously described (22,23).

Spontaneous hESC differentiation was induced as previously described (6,20). hPES-1 and hPES-2 cells at P60 and hPES-2′ cells at P70 were removed from the dishes using 1 mg/ml of collagenase IV (cat. no. 17104-019; Invitrogen/Gibco, Grand Island, NY, USA) and cultured under suspension conditions. Spontaneous EBs were grown in medium that included 80% Knockout-DMEM (Cat. no. 10829-018), supplemented with 20% serum replacement, 0.1 mM 2-mercaptoethanal and 1% non-essential amino acids (Cat. no. 11140-050) (all from Invitrogen/Gibco). After 14 days, all differentiated samples from EBs were collected.

### Karyotyping

The hBES-1 (P19) colonies, hESCs and HFFs were incubated with 0.2 *μ*g/ml of colchicine (Invitrogen/Gibco) at 37°C for 3 h. The cells were collected, trypsinized, washed with phosphate-buffered saline (PBS) (Cat. no. 10010-049; Invitrogen/Gibco) and then incubated with 0.075 M potassium chloride at 37°C for 10 min. These cells were fixed with methanol:glacial acetic acid (1:3) 3 times and then dropped onto glass slides. Chromosome spreads were Giemsa-banded and photographed. Karyotypes were assessed using normal G-banding procedures and 50 metaphase II spreads were examined for each sample. A normal karyotype showed normal chromosome numbers and G-banding patterns in the spreads examined.

### Array-based comparative genomic hybridization (aCGH)

The hBES-1 (P42), hPES-1 (P70), hPES-2 (P20) and hPES-2 (P55) cell colonies were lysed, after which genomic DNA was amplified using a SurePlex DNA Amplification system (BlueGnome Ltd., Cambridge, UK), according to the manufacturer’s instructions. Whole genome amplification (WGA) products were processed as previously described (24) according to the BlueGnome protocol (available at: http://www.cytochip.com/). Briefly, WGA products were fluorescently labeled and competitively hybridized to 24sure V3/24sure + arrays with matched control SureRef reference DNA (male/female) (both from BlueGnome Ltd.) in an array CGH experiment format. A laser scanner, InnoScanw 710 AL (Innopsys, Carbonne, France), was used to excite the hybridized fluorophores of a Fluorescent Labelling system (BlueGnome Ltd.), and used to read and store the resulting hybridization images. The scanned images were then analyzed and quantified using algorithm fixed settings with BlueFuse Multi software (BlueGnome Ltd.), a software package that automatically performed the steps of grid placement, quantification, normalization and post-processing.

### Flow cytometry for hESC phenotyping

The hBES-1 (P70) and hPES-2 (P70) cell colonies were digested with 0.25% Trypsin-EDTA (Cat. no. 15400-054; Invitrogen/Gibco) to prepare single cells. The hESCs (1−2×10^6^) were suspended in 0.5 ml of Dulbecco’s phosphate-buffered saline (DPBS; Cat. no. 14190-144; Invitrogen/Gibco). Subsequenlty, 2–3 ml of 70% ethanol were added to each tube, mixed and incubated at 4°C for 30 min. Cell suspensions were washed, centrifuged and resuspended in 400 *μ*l of DPBS. Cell suspensions were filtered, 1 mg/ml of propidium iodide (Asegene, Guangdong, China) was added followed by incubation for 30 min. Cell suspensions were analyzed using a FACSCalibur Flow Cytometer with CellQuest software (Becton-Dickinson, Bergen County, NJ, USA).

### DNA fluorescence in situ hybridization (FISH)

For DNA FISH analysis, the hBES-1, hPES-1 and hPES-2 cells were dropped onto wet slides and dried at room temperature overnight. They were then fixed with 0.1% Tween-20 and 0.01 N HCl and dehydrated with an ethanol series at concentrations of 70, 85 and 100%. CEPX/CEPY (Cytocell, Banbury, Oxfordshire, UK) was used for hybridization for 2 h. Finally, the cells were stained with 4′,6-diamidino-2-phenylindole (DAPI; Cytocell) for 5 min. The cells were examined under a fluorescence microscope (Leica DMIRE 2; Leica Microsystems GmbH, Wetzlar, Germany). At least 20 cells were examined for each experiment.

### RNA FISH

The hESCs, hBES-1 (P20 and P40), hPES-1 (P20 and P40), hPES-2 (P20 and P40) and hPES-2′ (P70) cells were used for RNA FISH analysis. XIST RNA-FISH (Biosearch Technologies, Novato, CA, USA) was carried out according to the protocol provided by Stellaris FISH Probes (Biosearch Technologies; https://www.biosearchtech.com/display.aspx?catid=224%2c318), as previously described (15). The XIST probe sequences are listed in Table I. hES cell colonies and hESCs were cultured on Millicell EZ slides (Cat. no. PEZGS0496; Millipore, Billerica, MA, USA). Stained colonies were examined under a fluorescence microscope (Leica DMIRE 2; Leica Microsystems GmbH).

### Immunofluorescence staining

The hESCSs, hBES-1 (P20 and P40), hPES-1 (P20 and P40), hPES-2 (P20 and P40), and hPES-2′ (P70) cells were used for immunofluorescence staining. The hES cell colonies and hESCs were cultured on Millicell EZ slides (Millipore) and fixed with 4% paraformaldehyde (Cat. no. P6148) for 20–30 min, treated with 0.5% Triton X-100 (Cat. no. X100) for 20 min and then blocked with 10% goat serum (Cat. no. G9023) (all from Sigma, St. Louis, MO, USA) for 1 h. The cells were then incubated with primary antibodies at 4°C overnight. The primary antibodies included mouse anti-histone H3 trimethyl K27 (H3K27me3; 1:100; Cat. no. ab6147) and rabbit anti-histone H3 acetyl K9 (H3K9ac; 1:200; Cat. no. ab61231) (both from Abcam, Cambridge, UK). The cells were then rinsed 3 times with PBS and incubated at 37°C for 60 min with goat anti-mouse IgM R-PE (1:200; Cat. no. 488800A; Invitrogen, Carlsbad, CA, USA) or goat anti-rabbit IgG FITC (1:200; Cat. no. A24532; Invitrogen), and finally stained with DAPI for 5 min. The stained colonies were examined under a fluorescence microscope (Leica DMIRE 2; Leica Microsystems GmbH).

### Real-time polymerase chain reaction (PCR)

XIST expression (probe XIST ID: Hs01079824_m1; Ambion, Austin, TX, USA) was assessed in the hESCs and HFFs, the hBES-1, hPES-1 and hPES-2 cells at P20, P40 and P60, the hPES-2′ undifferentiated cells at P50, P60 and P70, as well as in EBs of hPES-1 (P60), hPES-2 (P60) and hPES-2′ (P70) cells. X-linked gene expression was assessed in the hBES-1, hPES-1 and hPES-2 cells at P20, P40 and P60, and in the hPES-2′ undifferentiated cells at P50, P60 and P70. Real-time PCR was carried out using TaqMan gene expression Cells-to CT kits (Cat. no. 4399002; Ambion) as described in the TaqMan gene expression Cells-to CT kit protocol (http://www.lifetechnologies.com/order/catalog/product/4399002?ICID=search-product). The target X-linked genes were alpha thalassemia/mental retardation, X-Linked (ATRX; assay ID: Hs00230877_m1) and cysteine-rich hydrophobic domain 1 (CHIC1; assay ID: Hs01371424_m1) (both from Ambion). Relative XIST gene and other X-linked gene expression levels were calculated using the 2^−∆∆CT^ method following normalization to GAPDH (assay ID: Hs02758991_m1; Ambion) expression levels.

### Pluripotent characterizations of hPES-2 cells

To exclude the possibility that XIST expression in undifferentiated hPES-2 cells occurred due to their differentiation in culture, we assessed the pluripotent characterizations of the hPES2 cells. EBs were used to assess the differentiation capability of the hPES-2 cells *in vitro* using specific immunofluorescence staining.

Alkaline phosphatase (AP) activity was assessed by histochemical staining. The hPES-2 cell colonies on a mouse embryonic fibroblast (MEF) feeder layer were fixed with 4% paraformaldehyde for 20–30 min, treated with 0.5% Triton X-100 for 10 min and then stained with BCIP/NBT (Beyotime, Haimen, Jiangsu, China) for 5–13 min prior to examination.

Briefly, for immunofluorescence staining, the hPES-2 cell colonies were incubated with the following primary antibodies against stage-specific embryonic antigens: rat anti-human stage-specific embryonic antigen (SSEA)3 monoclonal antibody (Cat. no. LV1528429), rat anti-human SSEA4 monoclonal antibody (Cat. no. LV1488380), mouse anti-human tumor-rejection antigen (TRA)-1-60 monoclonal antibody (Cat. no. LV1541028), mouse anti-human TRA-1-81 monoclonal antibody (Cat. no. LV1580855); mouse anti-human octamer-binding transcription factor 4 (OCT-4) monoclonal antibody (Cat. no. MAB4419A4) and mouse anti-human Nanog homeobox (NANOG) monoclonal antibody (Cat. no. MABD24A4) (all from Millipore) (all were used at 1:100). The secondary antibodies were as follows: goat anti-rat IgM 488 (Cat. no. 549138), goat anti-mouse IgM R-PE (Cat. no. 488800A) (both from Invitrogen), goat anti-rat IgG, FITC (Cat. no. CW0167) and goat anti-mouse IgG, FITC (Cat. no. CW0113) (both from CWBIO, Beijing, China) (all were used at 1:200).

Gene expression levels in the pluripotent hPES2 cells were assessed by real-time PCR. The primers used for OCT-4, REX1 [also referred to as zinc-finger protein-42 (ZFP42)], SRY (sex determining region Y)-box 2 (SOX2), NANOG, Lin-28 homolog A (LIN28) and nucleophosmin (NPM1) are listed in Table II. β-actin was used as a control. PCR products were size-fractionated by 1% agarose gel electrophoresis and were visualized by ethidium bromide staining. Final analysis was made using an image analyzer (Bio-Rad, Hercules, CA, USA).

### Statistical analysis

The results from real-time PCR for the expression levels of the different genes in the different cell lines were compared by ANOVA. Pearson correlation coeffi-cients were determined to assess possible associations between XIST RNA and X-linked gene expression levels. A P-value of <0.05 was considered to indicate a statistically significant difference.

## Results

### Karyotype instability in hPES cell lines

**Figure 1:**
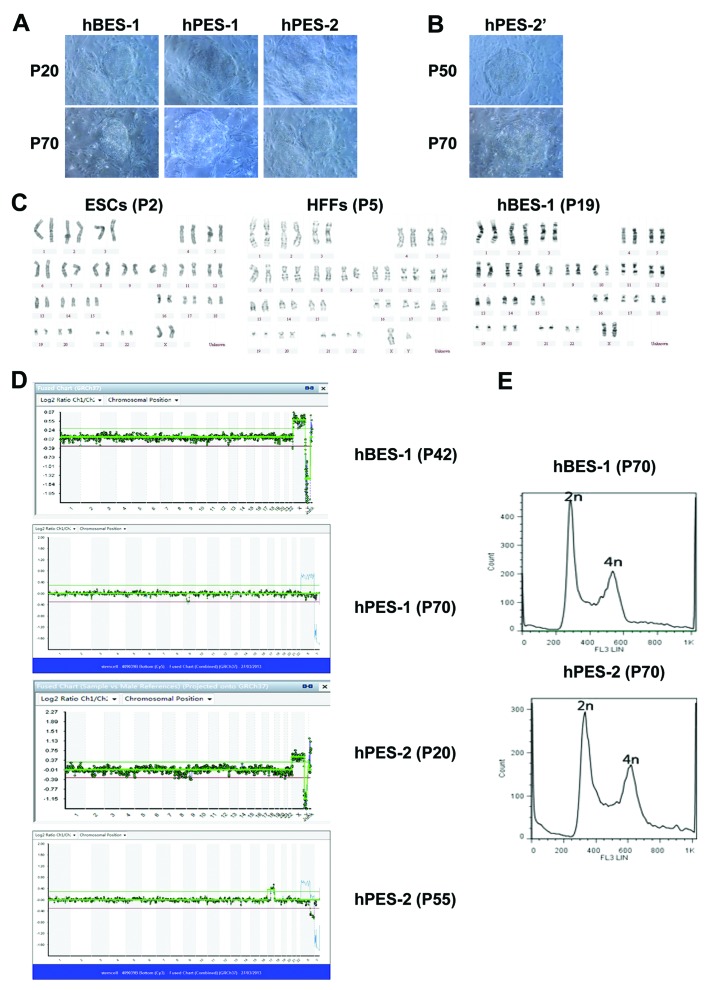
Human embryonic stem (hES) cell line morphologies and karyotypes. (A) Morphologies of human biparental embryonic stem cell line-1 (hBES-1), human parthenogenetic embryonic stem cell line-1 (hPES-1) and hPES-2 cell colonies at passage (P)20 and P70 as observed under an inverted microscope. (B) Morphologies of hPES-2′ cell colonies at P50 and P70 as observed under an inverted microscope. (C) Karyotypes. Endometrial stromal cells (ESCs) at P2 (positive control): normal 46,XX; human foreskin fibroblasts (HFFs) at P5 (negative control): normal 46,XY; and hBES-1 cells at P19: normal 46,XX. (D) Array-based comparative genomic hybridization (aCGH). hBES-1 cells (P42): normal 46,XX; hPES-1 cells (P70): normal 46,XX; hPES-2 cells (P20): normal 46,XX; and hPES-2 cells (P55): X microdeletion [XX,del(X)(q22.3;q28),+(17)(q121.31;q25.3)]. (E) hBES-1 and hPES-2 cell DNA contents as assessed by FACS; all were diploid.

The hBES-1, hPES-1 and hPES-2 are hES cell lines that have been strictly validated. The hBES-1 cell line is the parent source of hES cell lines, and hPES-1 and hPES-2 cells are well known parthenogenetic embryonic stem cell lines (6,20). The hBES-1, hPES-1 and hPES-2 cells had stabilized at >70 generations when cultured under identical culture conditions. The hBES-1, hPES-1 and hPES-2 cells retained the unique morphological and growth characteristics of hESCs (Fig. 1A and B).

We found that the chromosome karyotypes of hPES-2 cells changed during their long-term culture *in vitro*. At P20 (early generations), the results from aCGH for the hPES-2 cells were 46,XX. At P55, X chromosome microdeletions were observed in the hPES-2 cells, and the aCGH results were 46,XX,del(x) (q22.3;q28),+(17)(q121.31;q25.3) (Fig. 1C–E). In our previous study [Mai *et al* (6)], we reported X chromosome microdeletions in the same hPES cell lines. These phenomena indicated that the hPES-2 cell chromosomes were unstable and that their karyotypes could change during long-term culture. These phenomena did not occur in the hBES-1 and hPES-1 cell lines (Table III).

### Three XCI statuses identified in hPES cells

XIST RNA and H3K27me3 accumulation on X chromosomes has been reported to be a sign of XCI, accompanied by the loss of H3K9ac expression at H3K27me3 accumulation sites (25–31). In this study, we used XIST RNA, H3K27me3 and H3K9ac as indicators of XCI.

**Figure 2:**
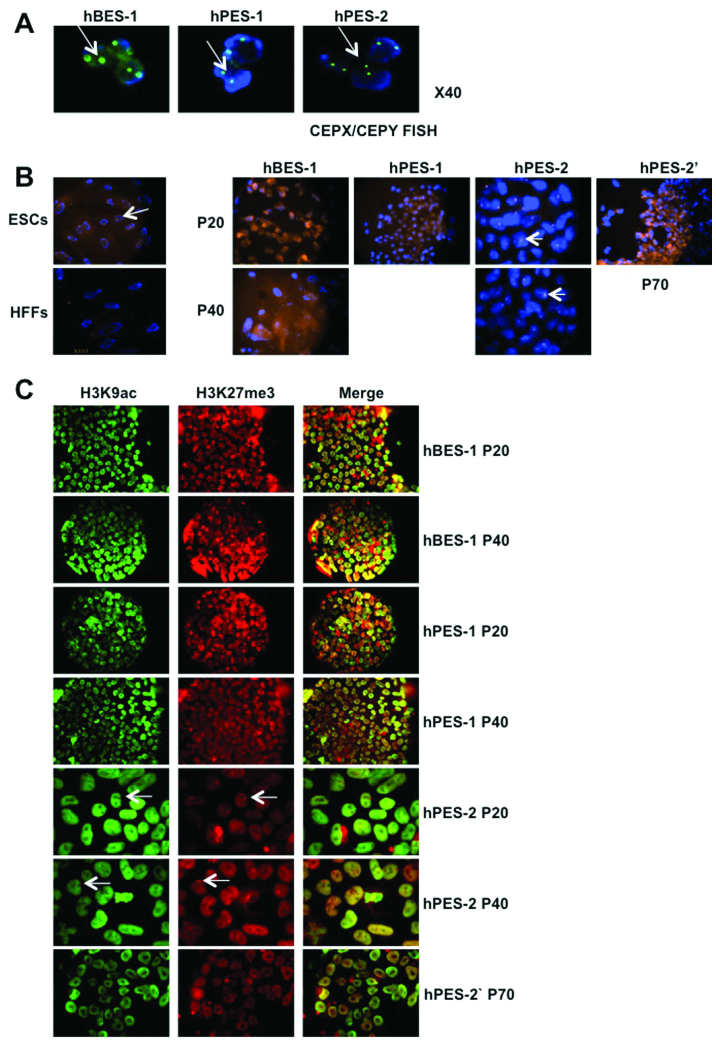
X chromosome inactivation (XCI) statuses of human biparental embryonic stem (hBES) and human parthenogenetic embryonic stem (hPES) cell lines. (A) CEPX/CEPY FISH analysis for hBES-1, hPES-1 and hPES-2 cells: red and green spots indicate chromosomes Y and X, respectively. All these human ES cells had 2 X chromosomes with no Y chromosome (white arrow). (B) X-inactive specific transcript (XIST) RNA FISH: endometrial stromal cells (ESCs) and hPES-2 cells: XIST RNA FISH signal (orange color and white arrow) showed XIST RNA coating on Xi; human foreskin fibroblasts (HFFs), hBES-1, hPES-1 and hPES-2′ cells: no XIST RNA FISH signals. (C) H3K9ac/H3K27me3 immunofluorescence. hPES-2 cells: immunostaining results with antibodies against H3K27me3 (red color and white arrow) coated on Xi without H3K9ac (green color) coat. hBES-1, hPES-1 and hPES-2′ cells: no H3K27me3 signals were detected in the cells. Punctate XIST FISH signals and foci of H3K27me3 without H3K9ac staining indicated the presence of Xi.

**Figure 3:**
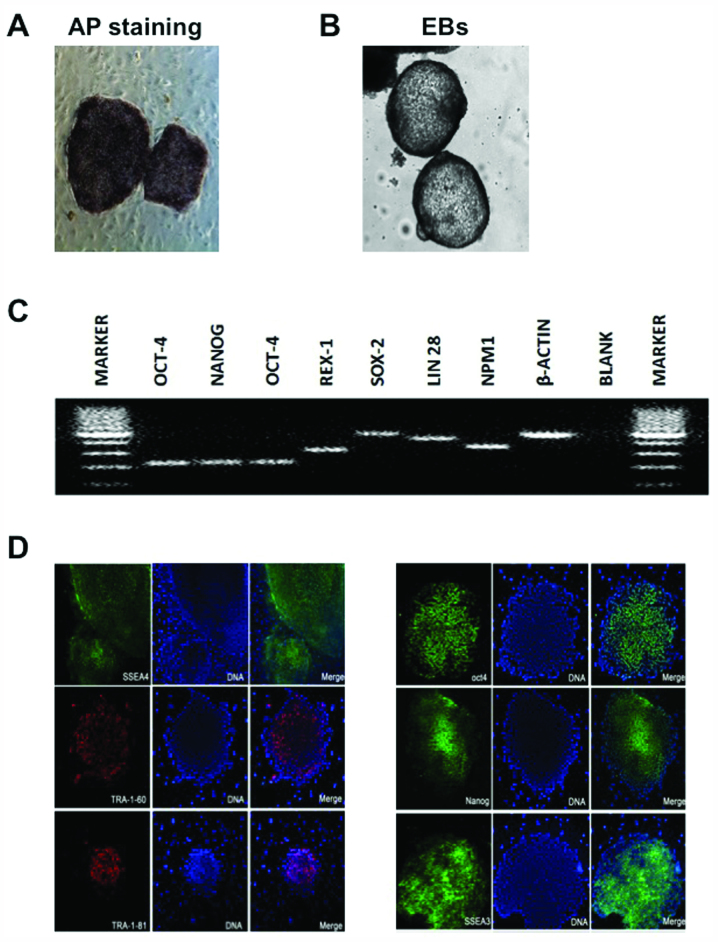
Pluripotentiality of human parthenogenetic embryonic stem cell line-2 (hPES-2). (A) Alkaline phosphatase (AP) staining, (B) embryoid bodies (EBs), (C) pluripotency gene expression, including octamer-binding transcription factor 4 (OCT-4), REX1 [also referred to as zinc-finger protein-42 (Zfp42)], SRY (sex determining region Y)-box 2 (SOX2), Nanog homeobox (NANOG), Lin-28 homolog A (LIN28) and nucleophosmin (β-actin was used as a control), amplified by real-time PCR, and (D) pluripotency immunofluorescent markers, including stage-specific embryonic antigen (SSEA)3, SSEA4, tumor-rejection antigen (TRA)-1-60, TRA-1-81, OCT-4 and NANOG.

**Figure 4:**
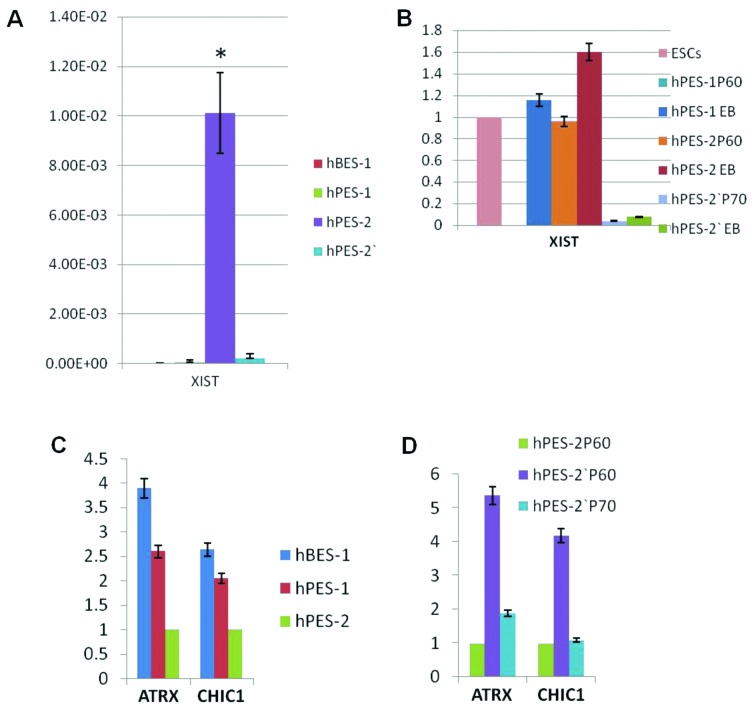
Gene expression. (A) Average X-inactive specific transcript (XIST) RNA expression levels in human biparental embryonic stem (hBES) and human parthenogenetic embryonic stem (hPES) cell lines. The average XIST RNA expression was higher in the hPES-2 cells (P<0.001, n=3). (B) Relative XIST RNA expression levels in hPES-1 [passage (P)60], hPES-2 (P60), and hPES-2′ (P70) cells and their embryoid bodies (EBs). Relative XIST RNA expression in EBs of hPES-1 and hPES-2 cells was >5-fold higher compared to the EBs of hPES-2′ cells [normalization to endometrial stromal cells (ESCs)]. (C) Average relative expression levels of alpha thalassemia/mental retardation, X-Linked (ATRX) and cysteine-rich hydrophobic domain 1 (CHIC1_in hBES and hPES cells. Average relative expression levels of ATRX and CHIC1 in hBES-1 and hPES-1 cellls was >2-fold higher than that in hPES-2 cells (n=3, normalization to hPES-2 cells). (D) Relative expression levels of ATRX and CHIC1 in hPES-2 and hPES-2′ cells. Relative expression levels of ATRX and CHIC1 in hPES-2′ cells at P60 were >2-fold higher than that in hPES-2′ cells at P70 of which were similar to hPES-2 cells at P60 (normalization to hPES-2 cells at P60). Statistical comparisons were made by one-way ANOVA. ^*^P<0.05 indicates significant difference.

We assessed the XCI status of hBES-1, hPES-1 and hPES-2 cell clones at P20 and P40, and found that XIST RNA and H3K27me3 did not accumulate in the hBES-1 and hPES-1 cells, which suggested that early-passage hBES-1 and hPES-1 cells did not undergo XCI. Subsequently, we further verified the X chromosome contents in the hBES-1, hPES-1 and hPES-2 cells using CEPX/CEPY FISH. These 3 hES cell lines all contained 2 X chromosomes (Fig. 2A). However, XIST RNA and H3K27me3 accumulation and H3K9ac loss were observed in the hPES-2 cells at P20, which suggested that XCI had been activated during early passage and maintained in the hPES-2 cells at P40 (Fig. 2B and C, Table IV). A previous study suggested that XCI in mouse embryonic stem cells indicated cell differentiation (32). However, in this study, the hPES-2 cells still expressed high levels of pluripotency genes and proteins and formed EBs *in vitro*, which suggested that the hPES-2 cells did not undergo differentiation (Fig. 3). Real-time PCR results also showed that XIST RNA expression was high in hPES-2 cells, but was very low or negative in hBES-1 and hPES-1cells (P<0.001) (Fig. 4A).

To validate the XCI status in the hPES-2 cells, hPES-2 cells at P45 (subclones of hPES-2 cells; designated as hPES-2′ cells) that had been stored in liquid nitrogen were recovered. These cells grew normally and passed to P70. However, these cells differed from the hPES-2 cells, as the XIST RNA expression levels in the hPES-2′ cells were very low (P<0.001; Fig. 4A); XIST RNA and H3K27me3 did not accumulate on X chromosomes (Fig. 2B and C, Table IV). In EB forming assays, the hPES-1 and hPES-2 EBs had a higher XIST RNA expression, whereas the hPES-2′ EB XIST RNA expression was extremely low (Fig. 4B).

All of these results indicated that there were different XCI statuses in the hPES cells: i) pre-XCI status: hPES-1 cells that did not express XIST RNA, and re-expressed XIST RNA following differentiation; ii) XCI status: hPES-2 cells expressed XIST RNA and this expression was sustained following differentiation; and iii) quiescent XCI status: low XIST RNA expression in hPES-2′ cells, which remained low following differentiation.

### X-linked gene expression levels in hPES cells

ATRX and CHIC1 are X-linked genes that are silenced during XCI. As expected, the ATRX and CHIC1 expression levels were very low in the hPES-2 cells, and higher in the hBES-1 and hPES-1 cells. The ATRX and CHIC1 expression levels in the hPES-2′ cells at P60 were higher than those in the hPES-2 cells at P60, but were decreased in the hPES-2′ cells at P70 and were as low as in those in the hPES-2 cells at P60, suggesting that a low XIST RNA expression in the hPES-2′ cells at P70 had occurred (Fig. 4C–D).

**Figure 5:**
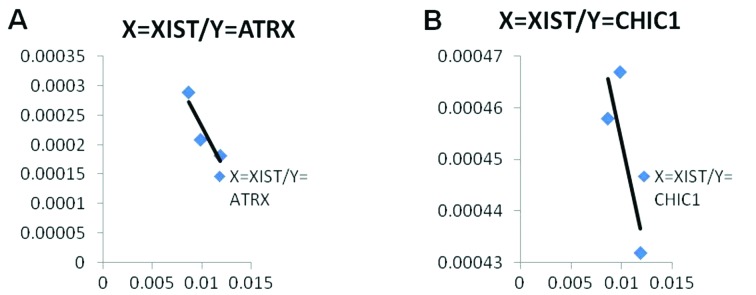
A tendency for a negative correlation between X-linked gene and X-inactive specific transcript (XIST) RNA expression levels, but no statistically significant difference was observed in the human parthenogenetic embryonic stem cell line-2 (hPES-2). (A) alpha thalassemia/mental retardation, X-Linked (ATRX) and XIST expression: R^2^=−0.91, P=0.268, n=3; (B) CHIC1 and XIST expressions: R^2^=−0.81, P=0.402, n=3.

The results from a correlation analysis suggested that there was a tendency for a negative correlation between X-linked genes and XIST RNA expression levels, although this was not statistically significant (P>0.05; n=3). An increased sample size may have provided a significant result. However, this still indicated that XIST RNA mediated the silencing of these X-linked genes (Fig. 5).

## Discussion

Using hPES cells may be a means to resolve human embryo stem cell transplantation immune rejection issues (33,34), although the security of their application remains a concern. Studies have found that human and murine parthenogenetic embryonic stem cells have genetic instabilities and epigenetic abnormalities (33,35). Thus, it is essential to understand the genetic and epigenetic characteristics of hPES.

In the current study, we found that some hPES cell lines had chromosome karyotype instabilities. The hPES-1 cells had a stable karyotype following serial passage, whereas the hPES-2 cells lost some X chromosome fragments after 50 passages. Previous studies have demonstrated that parthenogenetic embryonic stem cells often undergo changes associated with karyotype abnormalities (33). Liu *el al* (36) also reported X chromosome losses in hPES cells. These, as previously suggested, the genetic characteristics of hPES cells would not be stable following long-term culture (37).

To date, 3 XCI statuses have been reported for hES cells. With status I in hES cells, 2 X chromosomes are both activated in the undifferentiated stage, and XCI occurs randomly following differentiation, similar to what occurs in mouse embryonic stem cells (15–17). With status II, XCI has already occurred in undifferentiated hES cells, and approximately 20–70% of hES cells will have XIST clouds accumulated on specific chromosomes (11,18). With status III, XCI has occurred without XIST RNA expression (11). The XCI status may be associated with the genetic characteristics of the cells themselves, although it may also be affected by the culture conditions used. Some investigators have assumed that XCI in hES cells moved from state I to a transition state II to III, as an adaptation to the environmental conditions *in vitro* (11,18). Lengner *et al* (19) found that blastocyst-derived hES cells did not have XCI when cultured under low oxygen concentrations (5%); this status was associated with the pluripotency of stem cells and was affected by the oxygen concentration and atmospheric pressure. These data indicate that the culture conditions used, the methods used to establish cell lines, as well as other factors affect the XCI status of hES cells.

In the present study, we also found 3 XCI statuses that respectively matched those of hES cells: i) pre-XCI status, which was similar to hES status I: hPES-1 cells did not express XIST RNA, but did express X-linked genes, and re-expressed XIST RNA following differentiation, hPES-1 cells for example; ii) XCI status, which was similar to hES status II: hPES-2 cells expressed XIST RNA and expressed X-linked genes at a very low level, and XIST RNA expression was sustained following differentiation, hPES-2 cells for example; and iii) quiescent XCI status, which was similar to hES status III: low XIST RNA and X-linked gene expression in hPES-2′ cells, and XIST RNA expression remained at a low level following differentiation, hPES-2′ cells for example. The results observed for the hPES-2′ cells may have been due to their development from hPES-2.

The hES cells used in this study all underwent freezing and thawing and long-term culture *in vitro*. A previous study suggested that the freezing process and the *in vitro* culture conditions may alter the epigenetic state of stem cells (19). In this study, unstable XCI was found in the hPES-2 cells, and stable XCI (XCI I) was found in the hPES-1 and hBES-1 cells, which was similar to the results from the study by Liu *et al* (38). Unstable XCI may have been associated with chromosome instability in the hPES-2 cells. Unstable chromosomes rendered this cell line susceptible to environmental conditions and the freezing process used, which may have been the result of a environmental adaptations.

There is no paternal genetic material in parthenogenetic embryonic stem cells, and the 2 X chromosomes are all from the mother. The XCI status of hPES cells that lack paternal genetic material seems to be similar to that of embryonic stem cells. Liu *et al* (36) found that early-passage hPES cells did not exhibit XCI, but that XIST RNA expression and the XCI status emerged slowly during the course of long-term culture *in vitro*. Others have found that different hPES cells lines have different XCI states, which was similar to the situation with hES cells (38). To the best of our understanding, the XCI status of hES cells and the factors that affect it are more significant.

In conclusion, our data demonstrated that the chromosome karyotypes of some hPES cell lines exhibited instabilities. Similar to the hES cells, the hPES cells had 3 XCI statuses. Unstable XCI of hPES-2 cells may be related to chromosome instability. Unstable chromosomes render this cell line susceptible to environmental conditions and the freezing process used, which may be the result of an environmental adaptation. XCI plays important roles in sustaining embryo formation and cell biological activity (14), and an abnormal XCI can result in some diseases (39). Thus, it is essential to routinely assess the XCI status of hES cells, including hPES cells prior to their use in clinical applications.

## Figures and Tables

**Table I:** XIST probe sequences.

Probe sequence (5′→3′)	Probe sequence name
gaattgcagcgctttaagaactgaagg	Human XIST-RNAFISHprobe_1
gagagagtaagaaatatggctgcagca	Human XIST-RNAFISHprobe_2
gacgtgtcaagaagacactaggagaaa	HumanXIST-RNAFISHprobe_3
gaagggaatcagcaggtatccgatacc	Human XIST-RNAFISHprobe_4
gatattccagagagtgcaacaacccac	Human XIST-RNAFISHprobe_5
cttagcttaactgcagagtcattctct	Human XIST-RNAFISHprobe_6
ccgagttatgcggcaagtctaaaatgg	Human XIST-RNAFISHprobe_7
tgcctgacctgctatcatccatcttgc	Human XIST-RNAFISHprobe_8
ttagctcatgcaatgcacatgacttcc	Human XIST-RNAFISHprobe_9
cgatacaacaatcacgcaaagctccta	Human XIST-RNAFISHprobe_10
ccgcaatgtcaaaatcgccattttaag	Human XIST-RNAFISHprobe_11
cattttggacaacctaacaaagcacag	Human XIST-RNAFISHprobe_12
acttgaacactgcgacagaactggatc	Human XIST-RNAFISHprobe_13
catcttttcctgtgtgaccgcacatgt	Human XIST-RNAFISHprobe_14
catgttttacactgcggcaagaccttc	Human XIST-RNAFISHprobe_15
catatgacaacgcctgccatattgtcc	Human XIST-RNAFISHprobe_16
gatgtccacgtgacaaaagccatgata	Human XIST-RNAFISHprobe_17
ctctaattggctgtgatcaattccacc	Human XIST-RNAFISHprobe_18
gtgtgtcatcagtctaattccatcttc	Human XIST-RNAFISHprobe_19
gtgttcctcttgaggaaggcaggaatt	Human XIST-RNAFISHprobe_20
tcagtactgaagatcagcaatgccaag	Human XIST-RNAFISHprobe_21
cagagtgctgtctaatccaatgggtag	Human XIST-RNAFISHprobe_22
cgactggtagtcttcatgattaatggg	Human XIST-RNAFISHprobe_23
ctctaagaatgagtcagtcccactgct	Human XIST-RNAFISHprobe_24
aaggtggtaggtagttcacactatcta	Human XIST-RNAFISHprobe_25
aaggaaacttgggtagtcagaactcag	Human XIST-RNAFISHprobe_26
attgtagcgtgcaaataggatacagag	Human XIST-RNAFISHprobe_27
ctagtacagaggtcttgagtagtaagg	Human XIST-RNAFISHprobe_28
cactgctgaacactagggaagtgagtg	Human XIST-RNAFISHprobe_29
ctagtgcaaaggtcttgactagaggtc	HumanXIST-RNAFISHprobe_30
tagcactcctgctgctttgccaaggag	Human XIST-RNAFISHprobe_31
gcagtataagagaagaagcactagcta	Human XIST-RNAFISHprobe_32
agcgggattctactctaacataggggc	Human XIST-RNAFISHprobe_33
caagagagtgaattcaggctagttaga	Human XIST-RNAFISHprobe_34
tacttccagctgggatgtaaatacagt	Human XIST-RNAFISHprobe_35
caattacatgccatctacagttcgaag	Human XIST-RNAFISHprobe_36
gataggtcagaaacccaagtctaattg	Human XIST-RNAFISHprobe_37
ggccttaggtgtcaccaaccatgctgt	Human XIST-RNAFISHprobe_38
ctagtgcatagcaacctcgacaaatac	Human XIST-RNAFISHprobe_39
cagtgtgcgattacgcacataaatgtc	Human XIST-RNAFISHprobe_40
gagagtaggaccttattcacatggaat	Human XIST-RNAFISHprobe_41

XIST, X-inactive specific transcript.

**Table II:** Primers for pluripotency genes.

Gene	Forward (5′-3′)	Reverse (5′-3′)	Annealing temperature (°C)	Product size (bp)
OCT-4	GACAACAATGAGAACCTTCAGGAGA	TTCTGGCGCCGGTTACAGAACCA	55	218
NANOG	CAGAAGGCCTCAGCACCTAC	CTGTTCCAGGCCTGATTGTT	55	216
REX-1	GCGTACGCAAATTAAAGTCCAGA	CAGCATCCTAAACAGCTCGCAGAAT	58	306
SOX-2	CCCCCGGCGGCAATAGCA	TCGGCGCCGGGGAGATACAT	58	448
LIN28	AGTAAGCTGCACATGGAAGG	ATTGTGGCTCAATTCTGTGC	58	420
NPM1	TGGTGCAAAGGATGAGTTGC	GTCATCATCTTCATCAGCAGC	58	343
β-actin	CGGATGTCCACGTCACACTT	GTTGCTATCCAGGCTGTGGT	55	469

OCT-4, octamer-binding transcription factor 4; NANOG, Nanog homeobox; REX-1 [also referred to as zinc-finger protein-42 (Zfp42)]; SOX-2, SRY (sex determining region Y)-box 2; LIN28, Lin-28 homolog A; NPM1, nucleophosmin.

**Table III:** Karyotypes of human embryonic stem cells.

	Chromosome karyotyping	aCGH	FACS
hBES-1	46,XX (P19)	46,XX (P42)	Diploid (P70)
hPES-1	46,XX (P40) [6[	46,XX (P70)	N/A
hPES-2	46,XX,del(X)(q22;q24), del(1)(q21;q25) (P57) [6[	46,XX (P20) 46,XX,del(X)(q22.3;q28),+(17)(q121.31;q25.3) (P55)	Diploid (P70)

hBES-1, human biparental embryonic stem cell line-1; hPES-1, human parthenogenetic embryonic stem cell line-1; aCGH, Array-based comparative genomic hybridization; N/A, no available data.

**Table IV:** XIST RNA and H3K27me3 statuses of hES cell lines.

	XIST RNA		H3K27me3	
	P20	P40	P20	P40
hBES-1	−	−	−	−
hPES-1	−	−	−	−
hPES-2	+	+	+	+

	P70		P70	

hPES-2′	−		−	

XIST, X-inactive specific transcript; hES, human embryonic stem; hBES-1, human biparental embryonic stem cell line-1; hPES-1, human parthenogenetic embryonic stem cell line-1.
